# Near-Roadway Pollution and Childhood Asthma: Implications for Developing “Win–Win” Compact Urban Development and Clean Vehicle Strategies

**DOI:** 10.1289/ehp.1104785

**Published:** 2012-09-24

**Authors:** Laura Perez, Fred Lurmann, John Wilson, Manuel Pastor, Sylvia J. Brandt, Nino Künzli, Rob McConnell

**Affiliations:** 1 Swiss Tropical and Public Health Institute, Basel, Switzerland; 2 University of Basel, Basel, Switzerland; 3 Sonoma Technology, Inc., Petaluma, California, USA; 4 Spatial Sciences Institute, and; 5 Program for Environmental and Regional Equity, University of Southern California, Los Angeles, California, USA; 6 Resource Economics and Center for Public Policy and Administration, University of Massachusetts–Amherst, Amherst, Massachusetts, USA; 7 Department of Preventive Medicine, Keck School of Medicine, University of Southern California, Los Angeles, California, USA

**Keywords:** air pollution, asthma, burden of disease, children, compact urban growth, risk assessment, vehicle emissions

## Abstract

Background: The emerging consensus that exposure to near-roadway traffic-related pollution causes asthma has implications for compact urban development policies designed to reduce driving and greenhouse gases.

Objectives: We estimated the current burden of childhood asthma-related disease attributable to near-roadway and regional air pollution in Los Angeles County (LAC) and the potential health impact of regional pollution reduction associated with changes in population along major traffic corridors.

Methods: The burden of asthma attributable to the dual effects of near-roadway and regional air pollution was estimated, using nitrogen dioxide and ozone as markers of urban combustion-related and secondary oxidant pollution, respectively. We also estimated the impact of alternative scenarios that assumed a 20% reduction in regional pollution in combination with a 3.6% reduction or 3.6% increase in the proportion of the total population living near major roads, a proxy for near-roadway exposure.

Results: We estimated that 27,100 cases of childhood asthma (8% of total) in LAC were at least partly attributable to pollution associated with residential location within 75 m of a major road. As a result, a substantial proportion of asthma-related morbidity is a consequence of near-roadway pollution, even if symptoms are triggered by other factors. Benefits resulting from a 20% regional pollution reduction varied markedly depending on the associated change in near-roadway proximity.

Conclusions: Our findings suggest that there are large and previously unappreciated public health consequences of air pollution in LAC and probably in other metropolitan areas with dense traffic corridors. To maximize health benefits, compact urban development strategies should be coupled with policies to reduce near-roadway pollution exposure.

Local governments and metropolitan planning authorities in the United States are increasingly under pressure to reverse long-standing patterns of urban sprawl and pursue instead what is termed “compact development.” One of the major forces behind this push for denser living, more infill housing, and enhanced public transit is, in fact, driving: approximately 20% of U.S. carbon dioxide (CO_2_) emissions come from passenger vehicles (Ewing et al. 2008). Therefore, development patterns that limit urban sprawl and vehicle miles traveled (VMT) can have a major impact on reducing greenhouse gas (GHG) emissions.

California has sought to reduce GHG with its landmark 2008 Sustainable Communities and Climate Protection Act (SB375; State of California 2008). The act mandates that metropolitan planning authorities integrate land use and transportation planning—that is, reduce urban sprawl—to reduce VMT and GHG emissions. The legislation also offers developers incentives in the form of quicker environmental review if they pursue “transit priority residential” projects that place new homes closer to major transit stops and transportation corridors.

California envisions a policy framework that combines compact urban growth, reduced VMT, and the promotion of clean vehicles. However, compact urban development policies that increase the number of people living near major roadways with flat or increasing emissions, as may occur in growing cities in the developing world, may result in adverse health effects. Several studies have shown that on-road traffic emissions can be extremely high within 100–200 m of busy roads, where many residential areas are located (Zhou and Levy 2007). For example, particle number concentration was shown to decrease by 60–80% in the first 100 m downwind from a major freeway in Los Angeles, California (Zhu et al. 2002).

Recent reviews conclude that near-roadway traffic emissions may not only trigger asthma symptoms, but also contribute to the development of asthma in children (Anderson et al. 2011a, 2011b). Therefore, estimates of the burden of disease associated with air pollution need to be revised to account not only for asthma symptoms that are directly triggered by air pollution exposure, but also for symptoms that occur in children who developed asthma as a consequence of near-roadway exposure, including symptoms triggered by other exposures. Risk assessment methods have now been developed for this purpose (Künzli et al. 2008), and we have shown the substantial impact this has on the total burden of disease attributed to pollution (Perez et al. 2009). With traffic reduction the next policy frontier, measures that can cut the continuous growth in VMT should produce uniformly positive results—if they also reduce near-roadway pollution exposure.

To clarify the potential impact of such measures, we estimated the burden of childhood asthma morbidity attributable to near-roadway and regional air pollution in Los Angeles County (LAC), California, and the potential influence of different pollution reduction scenarios, including compact urban development, on the burden of disease.

## Methods

We used population-attributable fractions to quantify the impact of air pollution on asthma-related outcomes in LAC for year 2007 for children < 18 years of age. We followed an existing methodological framework (Künzli et al. 2008; Perez et al. 2009) that we adapted for this new study as summarized below.

To estimate the prevalence of asthma attributable to near-roadway pollution exposure, we used a concentration–response function (CRF) from the Children’s Health Study (CHS), a large population-based cohort in Southern California, in which living near major roadways, a proxy for traffic-related pollution exposure, was associated with increased prevalence of asthma (McConnell et al. 2006). Details on CHS study design and recruitment methods have been published previously (McConnell et al. 2006; Peters et al. 1999). To be consistent with the exposure assignment of the CRF study, we used the TeleAtlas MultiNet roads network (http://www.tomtom.com/en_gb/licensing/products/maps/multinet/) to map major LAC roads, defined as freeways, highways, or major arterial roads. These were then linked to census population data to derive the percentage of persons living within 75 m of these roads. For the present study we linked exposure to census population data given at the parcel level, which increased accuracy relative to linkage at the census block level used in a previous analysis (Perez et al. 2009). To be consistent with the prior CRF outcome definition, we used as background risk the asthma prevalence reported in the CHS (defined by use of controller medications in the previous year or lifetime asthma with any wheeze in the previous year or severe wheeze in the previous 12 months).

**Figure 1:**
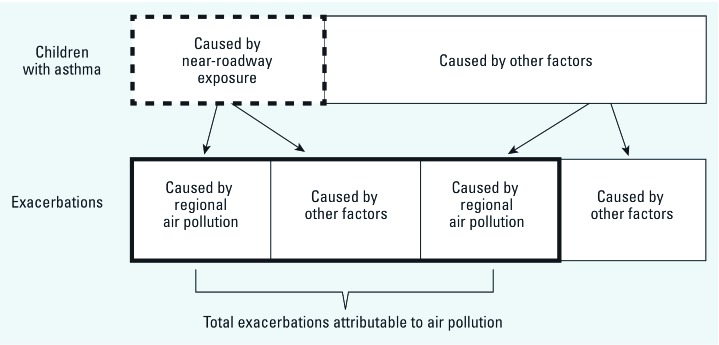
Conceptual model used to calculate asthma-related exacerbation attributable to air pollution for Los Angeles County based on Künzli et al. (2008). The thick dashed line indicates children with asthma attributable to near-roadway exposure. The thick solid line indicates total exacerbations due to regional and near-roadway air pollution.

Regional pollutants including particulate matter, nitrogen dioxide (NO_2_) and ozone (O_3_) are among the many causes of acute exacerbation among children with asthma, regardless of the cause of asthma onset (Jackson et al. 2011). However, an important consideration is that among those children with asthma attributable to living near a major road, all subsequent exacerbation should be attributed to air pollution, regardless of the trigger, assuming that these children would not otherwise have had the disease (Künzli et al. 2008). Conceptually, the total burden of asthma due to near-source and regional pollution includes the number of yearly asthma exacerbations triggered by causes other than regional air pollution among children whose asthma was caused (at least in part) by near-roadway pollution (Figure 1). These exacerbations are in addition to those directly triggered by regional air pollution exposure among all children with asthma, including children whose asthma was caused by near-roadway exposure and children whose asthma was caused by something other than traffic proximity. Air pollution risk assessments typically calculate only the asthma exacerbation burden triggered directly by regional pollution exposures, regardless of the underlying cause of asthma, whereas we included the additional burden of disease among children with asthma caused by near-roadway exposure but with exacerbations triggered by factors other than regional pollution.

To avoid double counting the burden associated with correlated regional pollutants, we estimated exacerbation attributable to NO_2_ or O_3_ only. NO_2_ was selected to represent urban-scale combustion-related pollution because it is correlated with particulate mass and other regional pollutants associated with respiratory health effects in southern California (Gauderman et al. 2004). O_3_ is produced as a result of photo-oxidation that is uncorrelated with other regional pollutants in the Los Angeles air basin (Gauderman et al. 2004).

The CRFs for bronchitis episodes among those with asthma, and for prevalent asthma attributable to near-roadway exposure, were derived from the CHS (McConnell et al. 2003, 2006) (Table 1). CRFs from appropriate studies of Southern California populations were not available for doctor visits, emergency department visits, or hospital admissions. Therefore, we applied CRFs used in a previous Southern California health impact assessment (Perez et al. 2009) or averaged the coefficient used in the previous analysis with the coefficient from a more recent study conducted in a similar population, as indicated in Table 1.

**Table 1:** Concentration–response functions (CRF) with 95% confidence intervals (CI) considered in the evaluation of air pollution burden.

Outcome	Estimate as published^a^ [transformed per 1 ppb]	Description (reference)
Asthma prevalence, near-roadway exposure				
Proximity (75 m) to major roadsb	1.64 (95% CI: 1.10, 2.44)	Age 5–7 years, Children’s Health Study (CHS) (McConnell et al. 2006)
Annual dispersion-modeled near-roadway NOx	2.07 (95% CI: 1.12, 3.83) per 11.6 ppb [1.065 (95% CI: 1.010, 1.123) per 1 ppb]	Same as above
Asthma exacerbation, regional pollutant exposure				
Bronchitis episodes				
NO2	1.070 (95% CI: 1.020, 1.130) per 1 ppb	24-hr average, age 9–13 years, CHS (McConnell et al. 2003)
O3	1.060 (95% CI: 1.000, 1.120) per 1 ppb	1000–1800 hours average, age 9–13 years, CHS (McConnell et al. 2003)
Doctor visits
NO2	1.061 (95% CI: 1.012, 1.113) per 24 ppb [1.0025 (95% CI: 1.0005, 1.0045) per 1 ppb]	24-hr average, age 0–14 years, London, UK (Hajat et al. 1999)
O3	1.054 (95% CI: 1.013, 1.096) per 50 ppb [1.0011 (95% CI: 1.0003, 1.0018) per 1 ppb]	24-hr average, age 2–14 years, Santiago de Chile (Ostro et al. 1999)
Emergency department visits				
NO2c	1.0011 (95% CI: 1.0002; 1.0021) per 1 ppb	Average of the two studies with risk estimate 1.026 (95% CI: 1.006, 1.049) per 27 ppb, 24-hr average, age ≤ 15 years, Europe (Sunyer et al. 1997); and OR = 1.027 (95% CI: 1.005, 1.05) per 20 ppb, 1 hr maximum, age 2–18 years, Atlanta, GA (Peel et al. 2005)c
O3	1.024 (95% CI: 1.015, 1.033) per 10 ppb [1.0024 (95% CI: 1.0015, 1.0033) per 1 ppb]	1 hr maximum, age 1–16 years, meta-analysis of 5 studies (Ostro et al. 2006)
Hospital admissions				
NO2	1.079 (95% CI: 1.054, 1.090) per 14 ppb [1.0054 (95% CI: 1.0038, 1.0038) per 1 ppb]	24-hr average, age ≤ 15 years, Hong Kong (Lee et al. 2006)
O3d	1.00240 (95% CI: 1.00161; 1.00317) per 1 ppb	Average of two following studies with risk estimate: 1.060 (95% CI: 1.041, 1.079) per 11.5 ppb, 8 hr mean, age ≤ 15 years, Hong Kong (Lee et al. 2006); 1.0175 (95% CI: 1.01, 1.0248) per 23 ppb, 8 hr maximum, age 0–17 years, New York State, respiratory disease including asthma (Lin et al. 2008)d
School absence for respiratory illness				
O3	1.829 (95% CI: 1.039; 3.22) per 20 ppb [1.031 (95% CI: 1.002, 1.060) per 1 ppb]	1000–1800 hours average, 4th grade, CHS (Gilliland et al. 2001)
aIn impact calculations, estimates were additionally corrected with the formula CRF/[1+It(CRF-1)], where It is the frequency of the outcome in the population (Zhang and Yu 1998). bDefined as functional road class (FRC) 01, FRC03, or FRC04 from TeleAtlas MultiNet roads network. cEstimate differs from that of Perez et al. (2009); derived with average of previously used and a newer study available for U.S. population (Peel et al. 2005). dEstimate differs from that of Perez et al. (2009); derived with average of previously used and a newer study available for U.S. population (Lin et al. 2008).				

The number of children < 18 years of age (> 2.5 million) was obtained from the American Community Survey (U.S. Census Bureau 2011). Background rates of the outcomes were obtained from the CHS or from local surveys (Table 2). Annual average daily concentrations of NO_2_ and O_3_ obtained from the 2007 U.S. Environmental Protection Agency Air Quality System (AQS) (U.S. Environmental Protection Agency 2009) and CHS monitoring stations were interpolated based on inverse distance-squared weighting to each census block group in the county to estimate population exposures. Because of the seasonality of school attendance and both the seasonal and day-of-week variability of O_3_, the O_3_ population exposure for school absences was based on 2007 daily maps, rather than annual maps, obtained from interpolated hourly ambient school-week concentrations projected to 2000 census block group centroids.

**Table 2:** Population size and baseline health outcome and exposure estimates used to evaluate the burden of asthma due to air pollution in LAC in 2007.

Variable	Value	Description (reference)
Target population
Total population of children, age 0–17 years	2,549,722	LAC, ages 0–17 population, 2007 (U.S. Census Bureau 2011)
Background level
Asthma prevalence in children	0.1257	CHS (McConnell et al. 2006)
Fraction reporting bronchitis symptoms (per year)	0.387	CHS (McConnell et al. 2003)
Fraction reporting doctor visits for asthma (per year)	0.751	CHS, personal communication, McConnell R, 2011a
No. of yearly emergency department visits for asthma (ICD-9: 493)	18,658	California breathing, Los Angeles County, 2007, personal communication, Milet M, 2011, based on California Office of Statewide Health Planning and Development, 2007
No. of yearly hospital admissions for asthma per year (ICD-9: 493)	3,131	California breathing, Los Angeles County, 2007, personal communication, Milet M, 2007, based on California Office of Statewide Health Planning and Development
Average daily school absence rate for respiratory illness among children with asthma	0.0158	CHS (Gilliland et al. 2001)
Population exposure (baseline)b
Proximity (75 m) to major roads (%)	17.8	Percent of LAC 2009 parcel population living within 75 m of nearest major roadc
Annual dispersion-modeled near-roadway NOx population-weighted concentration (ppb)	2.56	CALINE4, 2007, functional arterial classification code FCC3 (state highways)
Annual NO2 population-weighted concentration (ppb)	23.3	2007 U.S. EPA Air Quality System and CHS
8-hr maximum O3 population-weighted concentration (ppb)	39.3	2007 U.S. EPA Air Quality System and CHS
Abbreviations: CHS, Children Health Study; EPA, Environmental Protection Agency; ICD-9, International Classification of Diseases, 9th Revision (World Health Organization 1975). aFrom the CHS question “Ever been to doctor for wheezing?” among those that have ever had wheezing. This question was not restricted to the previous year. bRepresented by block group population-weighted concentration except for traffic proximity, which was represented by the average centroid parcel distance to busy roads. cDefined as functional road class (FRC) 01, FRC03, or FRC04 from TeleAtlas MultiNet roads network.				

We estimated that 17.8% of LAC children lived within 75 m of major roads, and that the annual population-weighted exposure to NO_2_ was 23.3 ppb (24-hr average) and to O_3_ was 39.3 ppb (8-hr maximum) in LAC (Table 2). We assumed background concentrations of 4 ppb for NO_2_ annually and 38 ppb for 8-hr maximum O_3_ on all days, based on long-term measurements (1994–2003) from CHS monitoring stations in clean coastal locations (i.e., Lompoc, CA) (McConnell et al. 2003). [The average population-weighted annual O_3_ for LAC was near background because population exposures in the areas with high O_3_ are offset by population exposures in areas with high oxides of nitrogen (NO_x_) emissions and very low O_3_ concentrations, due to nitric oxide (NO) in fresh vehicular exhaust scavenging O_3_ in those areas.] We considered three near-roadway proximity exposure reduction scenarios (Table 3):

**Table 3:** Exposure reduction scenarios for near-roadway exposure, regional NO_2_ and O_3_, and corresponding reduction in childhood asthma cases attributable to near-roadway pollution exposure (based on total of 320,500 children with asthma in LAC).

Scenarios	Change considered	Change in exposure from baseline	Hypothesized new population exposure	Change in prevalent cases^a^ (95% CI)		
				No.	Percent (95% CI)
Scenario 1 (reduction to background)										
Traffic proximity	–100%	–17.8%	0%	Decrease by 27,100 (4,900, 51,200)	8% (2%, 16%)
Dispersion-modeled near-roadway NOx	–100%	–2.56 ppb	0 ppb	Decrease by 39,800 (6900, 65,600)	12% (2%, 20%)
NO2	Decrease to background levels	–19.3 ppb	4 ppb	—	—
O3	Decrease to background levels	–3.03 ppb	36.3 ppb	—	—
Scenario 2 (reduced regional pollution and near-roadway exposure)										
Traffic proximity	–3.6%	–3.6%	14.2%	Decrease by 5,900 (1,000, 11,800)	2% (0.3%, 4%)
Dispersion-modeled near-roadway NOx	–20%	–0.51 ppb	2.05 ppb	Decrease by 8,400 (1,400, –14,300)	3% (0.4%, 4%)
NO2	–20%	–3.9 ppb	19.4 ppb	—	—
O3	–20%	–0.61 ppb	38.7 ppb	—	—
Scenario 3 (reduced regional pollution, increased near-roadway exposure)										
Traffic proximity	3.6%	3.6%	21.4%	Increase by 5,900 (1,000, 11,800)	2% (0.3%, 4%)
NO2	–20%	–3.9 ppb	19.4 ppb	—	—
O3	–20%	–0.61 ppb	38.7 ppb	—	—
aIncrease or decrease in asthma cases attributable to near-roadway pollution.

1. A reduction in annual concentrations of regional pollutants for each census block group to levels found in clean CHS communities (from 23.3 ppb to 4 ppb for NO_2_ and 39.3 ppb to 36.3 ppb for O_3_) in combination with a reduction in the proportion of children in the county living within 75 m of a major road from 17.8% to 0%
2. A 20% reduction in the annual concentrations of regional pollutants for each census block group (from 23.3 ppb to 19.4 ppb for NO_2_ and 39.3 ppb to 38.7 ppb for O_3_) in combination with a 3.6% reduction in the proportion of all children in the county living within 75 m of a major road (from 17.8% to 14.2%, corresponding to a 20% decrease in the proportion of children currently living within 75 m)
3. A 20% reduction in regional pollutant concentrations in combination with a 3.6% increase in the proportion of children living within 75 m of a major road (from 17.8% to 21.2%).

Scenario 1 reflects the total burden of preventable illness from both exposures. At this time there is considerable uncertainty regarding the potential impact of compact urban growth strategies on near-roadway exposures, so scenarios 2 and 3 were selected assuming moderate reductions in regional pollutants from continued regulatory efforts and a moderate 20% increase or decrease in near-roadway exposure—a value that was chosen for illustration and could be refined using data from regional planners as they become available. Regional pollutant concentrations aggregated to the census block group level that exceeded background levels were reduced linearly, whereas we assumed that concentrations at or below the background level would be unaffected by changes in emissions.

There are intrinsic limitations and uncertainties in risk analysis. We estimated a 95% confidence interval (CI) derived from the propagation of the CIs for the CRFs to address uncertainty in these estimates. In addition, proximity to major roadways has uncertainty as a proxy for near-roadway pollution exposure that depends on traffic volume, the emissions of the vehicular fleet, and local meteorological factors. Therefore, we also estimated the total burden of asthma-related exacerbations associated with a 100% and a 20% reduction in population-weighted exposure to the near-roadway dispersion-modeled pollution mixture (instead of a change in roadway proximity in exposure scenarios 1 and 2 in Table 3) using the CHS CRF from an estimate of the association of asthma prevalence with dispersion-modeled near-roadway pollution exposure accounting for traffic volume and emission factors (McConnell et al. 2006). Specifically, we used modeled NO_x_ to represent the incremental contribution of local traffic to a more homogeneous community background concentration of NO_x_ that included both primary and secondary pollution resulting from long-range transport and regional atmospheric photochemistry. It is a marker for correlated pollutants in the near-roadway mixture (rather than the etiologic agent for near-roadway health effects). We developed new estimates of population-weighted yearly average of local traffic-related NO_x_ concentrations for 2007 in LAC using the CALINE4 dispersion model with the 2007 TeleAtlas MultiNet Roadway network, and 2007 vehicle emission factors for Los Angeles from the EMFAC model (California Air Resources Board 2007). Vehicle emission factors were developed for winter (55^o^F/50% relative humidity) and summer (75^o^F/50% relative humidity) conditions using average speeds of 65 mph on freeways and highways [FCC (functional class code) 1 and FCC2 class roads], 50 mph on major arterials (FCC3 class roads), and 30 mph on minor arterials and collectors (FCC4 roads). The model used year 2000 traffic volumes adjusted to 2007 VMT provided by the California Department of Transportation (17.5% increase in VMT for LAC). Modeled NO_x_ concentrations were estimated for the block group centroids. The CHS CRF was developed for the contribution of local traffic on non-freeways using an older road functional roadway classification (FRC) scheme (McConnell et al. 2006) that is no longer available in a form that matches the most current FCC classification that we used. To minimize overestimation of population exposure to near-roadway exposure in LAC, we used estimates of exposure from FCC3 (major arterials) as representative of non-freeway roads used in developing the CHS CRF. We considered the impact of all near-FCC3 roadway NO_x_ (corresponding to a scenario of 100% reduction in modeled near-roadway pollution at the block group centroid) and of a 20% decrease in population exposure. This corresponds to the 3.6% reduction in the total population of children within 75 m of a major roadway (a 20% reduction the proportion of the total population living within 75 m) (Table 3).

## Results

Of the estimated 320,500 cases of childhood asthma in the county (based on Southern California prevalence of 0.1257) (Table 2), we estimated that approximately 27,100 (8%; 95% CI: 2%, 16%) were caused at least in part by residential proximity to a major road (Table 3). A 3.6% reduction in the proportion of children living within 75 m of a major road (scenario 2) would result in 5,900 fewer asthma cases (95% CI: 1,000, 11,800) caused by near-roadway exposure (2% of the total cases in the county; 95% CI: 0.3%, 4%), whereas a 3.6% increase (scenario 3) would result in an additional 5,900 asthma cases caused by near-roadway exposure (Table 3).

Estimates of yearly asthma-related exacerbations attributable to air pollution are presented in Table 4 for NO_2_ and O_3_, with results partitioned by cause of asthma (traffic proximity or other factors) using the conceptual scheme in Figure 1. Using this approach and assuming no near-roadway exposure and a reduction of NO_2_ or O_3_ to background levels (scenario 1), we estimated that 70,200 episodes (95% CI: 31,000, 95,700) of bronchitis (56.6% of all episodes) could be attributed to the combined effects of traffic proximity and regional NO_2_, our marker for the regional mixture of combustion-related pollutant exposure. The estimated burdens of other exacerbations attributable to exposure were smaller, ranging from 10.6% for emergency department visits to 19.5% for hospital admissions. The overall impact of air pollution was highly sensitive to the inclusion of exacerbations attributable to asthma triggers other than regional NO_2_ among children whose asthma was caused by traffic proximity. For example, we estimated that 65,100 bronchitis episodes were triggered by regional air pollution, including 5,600 episodes among children whose asthma was caused by near-roadway air pollution and 59,500 episodes among children whose asthma was caused by something other than air pollution (Table 4). In addition, we estimated that 5,100 bronchitis episodes (4.1% of the total) triggered by something other than regional air pollution would not have occurred if children had not lived within 75 m of a major road, because these children would not have developed asthma to begin with. These episodes would not have been accounted for if estimated effects of traffic proximity on asthma prevalence had not been considered. The estimated impact of such cases was especially large for outcomes that were weakly associated with regional NO_2_ (e.g., emergency department visits for asthma with an estimated CRF of 1.0011 per 1 ppb NO_2_) (Table 1), because exacerbations triggered by causes other than air pollution among children with asthma caused by traffic proximity account for a larger proportion of all episodes.

**Table 4:** Yearly number (%) of childhood asthma-related exacerbations attributable to near-roadway pollution in combination with regional NO_2_ and regional O_3_ above background levels in clean communities (scenario 1, traffic proximity model) (95% confidence intervals).^a^

Estimated no. of exacerbations (%)	Exacerbations due to regional air pollution among children with asthma caused by…			Exacerbations due to other causes among children with asthma caused by traffic proximity pollution	Total
	Traffic proximity pollution	Other factors	All causes		
NO2
Bronchitis episodes	124,034	5,600 (660, 12,100)	59,500 (20,500, 85,700)	65,100 (22,500, 92,800)	5,100 (900, 11,700)	70,200 (31,000, 95,700)
100%	4.5% (0.5%, 9.7%)	48.0% (16.5%, 69.1%)	52.5% (18.2%, 74.8%)	4.1% (0.7%, 9.4%)	56.6% (25.0%, 77.1%)
Hospital admissions	3,131	30 (5, 65)	340 (265, 420)	375 (295, 450)	235 (50, 450)	610 (410, 840)
100%	1.0% (0.2%, 2.0%)	10.9% (8.5%, 13.4%)	12.0% (9.4%, 14.4%)	7.6% (1.6%, 14.4%)	19.5% (12.9%, 26.7%)
ED visits	18,658	35 (5, 85)	370 (65, 670)	405 (75, 725)	1,570 (320, 2,970)	1,970 (690, 3,400)
100%	0.2% (0.0%, 0.5%)	2.0% (0.4%, 3.6%)	2.2% (0.4%, 3.9%)	8.4% (1.7%, 15.9%)	10.6% (3.7%, 18.2%)
Doctor visits	240,696	870 (70, 2,140)	9,200 (1,900, 16,500)	10,100 (2,000, 17,900)	19,800 (4,100, 37,700)	29,900 (12,300, 48,900)
	100%	0.4% (0.0%, 0.9%)	3.8% (0.8%, 6.8%)	4.2% (0.8%, 7.4%)	8.2% (1.7%, 15.7%)	12.4% (5.1%, 20.3%)
O3
Bronchitis episodes	124,034	1,610 (0, 4,050)	17,200 (530, 32,100)	18,800 (590, 34,900)	9,100 (1,900, 17,600)	27,800 (9,100, 44,300)
100%	1.3% (0.0%, 3.3%)	13.8% (0.4%, 25.9%)	15.1% (0.5%, 28.1%)	7.3% (1.5%, 14.2%)	22.4% (7.4%, 35.7%)
Hospital admissions	3,131	1.9 (0.3, 4.2)	20.7 (10, 31.7)	22.6 (10.9, 34.3)	270 (50, 510)	290 (80, 530)
100%	0.1% (0.0%, 0.1%)	0.7% (0.3%, 1.0%)	0.7% (0.3%, 1.1%)	8.5% (1.7%, 16.2%)	9.3% (2.4%, 16.9%)
ED visits	18,658	11 (2, 23)	121 (75, 167)	133 (84, 181)	1,590 (330, 3,020)	1,730 (460, 3,160)
100%	0.1% (0.0%, 0.1%)	0.6% (0.4%, 0.9%)	0.7% (0.4%, 1.0%)	8.5% (1.8%, 16.2%)	9.3% (2.5%, 16.9%)
Doctor visits	240,696	59 (6, 144)	632 (160, 1,111)	692 (175, 1,207)	20,600 (4,200, 39,100)	21,300 (4,800, 39,800)
100%	0.02% (0.002%, 0.1%)	0.3% (0.1%, 0.5%)	0.3% (0.1%, 0.5%)	8.6% (1.8%, 16.3%)	8.9% (2.0%, 16.5%)
Missed school days for respiratory diseases	1,350,391	27,900 (449, 70,600)	302,000 (43,800, 562,300)	329,900 (47,700, 612,100)	86,200 (12,000, 168,700)	416,100 (140,200, 681,500)
	100%	2.1% (0.0%, 5.2%)	22.4% (3.2%, 41.6%)	24.4% (3.5%, 45.3%)	6.4% (0.9%, 12.5%)	30.8% (10.4%, 50.5%)
ED, emergency department. aBased on traffic proximity as a proxy for near-roadway exposure effects (scenario 1); reduction in burden is represented by positive values.								

The estimate of the burden of bronchitis episodes directly attributable to O_3_ and traffic proximity pollution (18,790 preventable episodes triggered by regional air pollution under the usual risk assessment approach) was considerably less than for NO_2_ (65,100 episodes triggered by air pollution) (Table 4). O_3_ CRFs for outcomes other than bronchitis and missed school days were modest (e.g., for emergency department visits, 1.024 for each 10-ppb increase in O_3_) (Table 1); therefore, accounting for all exacerbations among children whose asthma was caused by traffic proximity led to substantial increases in estimates of disease burden attributable to O_3_—from 133 to 1,730 emergency department visits (Table 4).

**Figure 2:**
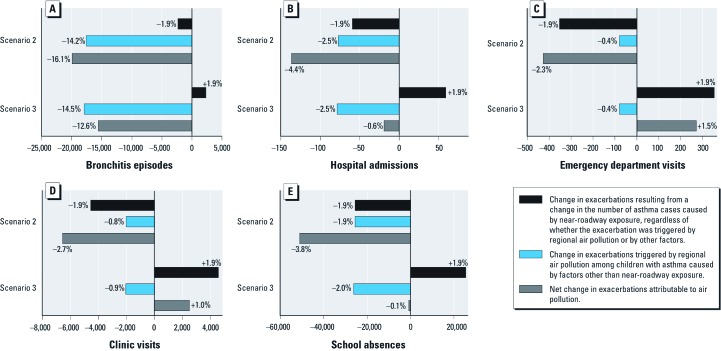
Number and percentage of exacerbations attributable to changes in pollutant levels under different exposure scenarios. Scenario 2 assumes a 3.6% decrease in children living near major roads and a 20% decrease in regional pollution. Scenario 3 assumes a 3.6% increase in children living near major roads and a 20% decrease in regional pollution. Regional pollution is represented by NO_2_ for all outcomes except O_3_ for school absences. Bars to the left and right of zero represent reductions and increases in the burden of asthma exacerbation, respectively, compared with baseline. (*A*) Bronchitis episodes, (*B*) hospital admissions, (*C*) emergency department visits, (*D*) clinic visits, and (*E*) school absences.

A partial reduction in the burden of asthma exacerbation could be achieved with a more modest reduction in population exposure to traffic proximity and regional pollutants. Figure 2 shows the estimated numbers of exacerbations (and percent of total) attributable to a 20% decrease in regional air pollution (either NO_2_ or O_3_) in combination with a 3.6% reduction in the proportion of all children living near major roadways (scenario 2) or a 3.6% increase in traffic proximity that might result from compact urban development (scenario 3). We report the estimated impacts for the regional pollutant with the strongest association with each outcome—based on a reduction in NO_2_ for all outcomes except school absences, which were estimated for a reduction in O_3_. The net impact of each air pollution reduction scenario depended on the total number of outcomes per year and the strength of the CRF for the regional pollutant. Thus, a 3.6% reduction in the proportion of all children in the county who lived near major roadways (scenario 2) would result in a 1.9% decrease in each outcome in Figure 2 (corresponding to the reduction in cases of asthma caused by near-roadway exposure, regardless of whether the exacerbation was triggered by air pollution or by other factors). For bronchitis episodes, which are relatively common and have a large CRF for NO_2_ (1.070 per 1-ppb increase) (Table 1), we estimate that a total of 19,900 exacerbations (16.1% of all episodes annually) could be prevented by a 20% NO_2_ reduction, most of which (17,600 episodes, 14.2%) were attributable to the reduction of episodes triggered by regional pollution among children whose asthma was not caused by air pollution, rather than to bronchitis episodes among children whose asthma was caused by near-traffic pollution (scenario 2 in Figure 2). If NO_2_ were reduced by 20% but traffic proximity increased by 3.6% (scenario 3), we estimated a smaller reduction in total bronchitis episodes (15,580 episodes, 12.6% of the total). In contrast, for emergency department visits (with the weakest CRF for NO_2_, 1.0011 per 1-ppb increase), most of the benefit under scenario 2 resulted from the estimated reduction in the number of children with asthma caused by traffic proximity pollution, whereas an increase in the population living near major roadways under scenario 3 resulted in an increase in emergency department visits that exceeded the modest benefit from NO_2_ reduction, resulting in a net increase in total number of visits. Other outcomes demonstrated intermediate patterns for the relative impact of a reduction in regional pollution and a change in traffic proximity, with the net absolute impact depending also on the baseline frequency of the outcome in the population (Figure 2).

In additional analyses we used the CRF from a dispersion-modeled estimate of the effect of near-roadway pollutant mixture exposure on asthma (2.07; 95% CI: 1.12, 3.83 per 16 ppb) (McConnell et al. 2006) and an estimated annual average contribution of near-roadway pollution derived from major arterials expressed as 2.6 ppb of NO_x_, or 8% of all near-roadway pollution generated from freeways, highways, state highways, minor arterials, and collectors. We estimated that 39,800 cases of prevalent asthma (12% of the total; 95% CI: 2%, 20%) were caused by near-roadway pollution based on this analysis, compared with 27,100 cases based on roadway proximity (scenario 1) and that a 20% decrease in near-roadway exposure (scenario 2) would result in 8,400 fewer cases (3% of all cases in the county; 95% CI 0.4%, 4%) compared with 5,900 fewer cases based on roadway proximity (Table 3). The total burden of asthma-related exacerbation attributable to the combination of near-roadway and regional pollution under these scenarios is shown in Supplemental Material, Tables S1 and S2 (http://dx.doi.org/10.1289/ehp.1104785). Although the numbers of exacerbations attributed to near-roadway exposure were increased, estimates were of the same order of magnitude as those obtained using traffic proximity as an indicator of near-roadway exposure.

## Discussion

The implications of near-roadway exposures for the burden of disease due to air pollution have not been fully appreciated. Our results indicate that risk assessment focusing exclusively on regional pollutant effects substantially underestimates the impact of air pollution on childhood asthma because it does not account for exacerbations caused by exposures other than air pollution among the approximately 8% of children with asthma in Los Angeles County whose asthma can be attributed to pollution from near-roadway pollution exposure based on proximity. Moreover, the burden of asthma exacerbation among children whose asthma was caused by living near roadways, and the potential benefits of reducing near-roadway exposures, are disproportionately larger for more severe and more expensive outcomes, such as hospital admissions and emergency department visits.

The use of traffic proximity is both a strength and limitation. A traffic proximity buffer is appealing as a regulatory metric because it is precise and easily measured. However, reductions in traffic density and vehicular emissions are not reflected in this metric, so estimates of the burden of disease based on the proximity CRF (for the Los Angeles Basin) may not easily be generalized to other urban settings. Proximity is a proxy indicator of local traffic-related pollutants that vary by type of roadway, over time, and by region, and that also depend on the pollution reduction technologies and age of local vehicular fleets [Health Effects Institute (HEI) 2009]. Therefore, we conducted a sensitivity analysis based on exposure estimates derived from a dispersion model of near-roadway traffic-related pollution that integrated traffic density, emission factors, and meteorology. Results of this alternative approach demonstrated a pattern of preventable asthma burden for a 100% decrease in near-roadway pollution exposure that was generally consistent with that estimated for a 100% decrease in residential proximity to major roads, and estimates for a 20% decrease in near-roadway pollution that were consistent with estimates based on a 20% decrease in proportion of children currently living near major roads (corresponding to the 3.6% decrease from 17.8% to 14.2% of all children in the county). It is also possible that living near a major traffic corridor is associated with socioeconomic characteristics and mold or other substandard housing characteristics that could explain the association of traffic proximity with asthma in studies from which the CRFs were derived. However, markers for these characteristics did not confound this association (McConnell et al. 2006). The CHS has also shown that the association between traffic proximity and asthma is modified by genetic variants in plausible biological pathways, which would be difficult to explain based on confounding by socioeconomic status or related characteristics (Salam et al. 2007).

Compact urban development (scenario 3) that increases the number of children living near major roadways could limit the overall benefit of reduced exposure to regional pollution, especially for emergency department and clinic visits and for school absences, if not accompanied by substantial reductions in emissions and traffic volume along major traffic corridors. In practice, the CRF associated with near-roadway proximity may be reduced if overall reductions in vehicle miles traveled and emissions result in reduced air pollution exposures near roadways. We did not attempt to estimate a more informative association between dispersion-modeled exposure and illness under scenario 3 because data are not readily available to make such estimates. Realistic estimates under different SB375 compact urban development scenarios of the likely impact on traffic volume and emissions factors, in addition to changes in population distributions along major traffic corridors, are urgently needed to identify strategies that will optimize the benefits of compact urban development on both GHG emissions and on respiratory health. Strong “win–win” policies could result from compact growth and other strategies that increase the number of people living along busy roads, thereby reducing vehicle-miles traveled and regional air pollution, if coupled with reduction of traffic-related primary emissions such as ultrafine particles and black carbon. Promoting the rapid adoption of low- or zero-emission vehicles powered by carbon-neutral energy sources, and limiting residential development in buffer zones very near the largest roadways, are obvious examples of such strategies. Conversely, our findings suggest that a compact urban development plan that increases the proportion of children near major roadways, while simultaneously expanding or adding new traffic corridors to accommodate greater traffic volume, will increase rather than decrease the burden of air pollution–related asthma. Although such a scenario may be unlikely in California, in other regions in the world—particularly in developing countries—it is plausible.

There are uncertainties in our estimates related to the conceptual approach. Until recently, the causal association of asthma onset and near-roadway pollution—an important assumption underlying our analysis—was uncertain (Eder et al. 2006). However, a scientific consensus is emerging that the observed epidemiological associations of higher asthma rates along major roads are causal (Anderson et al. 2011a, 2011b; HEI 2009; Ryan and Holguin 2010; Salam et al. 2008). In addition, our approach assumes that removal or reduction of near-roadway pollutant exposure would reduce the number of children developing asthma (Künzli et al. 2008). Asthma is likely to develop as a consequence of multiple, potentially synergistic, risk factors, and we may have overestimated the benefit of exposure reduction if some of the cases of childhood asthma attributed to near-roadway pollution would have developed due to competing risk factors even if the children had not been exposed to traffic. However, an 8-year follow-up of a Dutch birth cohort showed that the association between incident asthma and soot exposure did not diminish during follow-up, as might have been expected if some of the soot-associated cases would have developed due to competing causes in the absence of soot exposure (Gehring et al. 2010). Further research is needed to determine the potential role of competing risks, but the current understanding of asthma etiology warrants a precautionary preventive approach to near-roadway pollution effects. Our conceptual model of effects of exposure reduction also does not address the time lag that might be required for health benefits to be achieved.

We have previously acknowledged uncertainties related to the extrapolation of CRFs across populations, which we have attempted to minimize by applying CRFs, asthma prevalence, and outcome frequency estimates for Southern California, when available (Künzli et al. 2008). Statistical uncertainty was estimated by calculating 95% CIs, which were relatively wide. We accounted for the propagation of random variation based on the observed distribution of chronic and acute CRF estimates, but mean estimates are not influenced by this uncertainty.

We may have underestimated the burden of disease by using NO_2_ as a surrogate for regional combustion pollutants including also particulate matter and other pollutants, without accounting for additive effects of different pollutants. We used spatial mapping of ambient daily air quality data to assign exposure to NO_2_ and O_3_ at the census block level. Although the accuracy of assignment from the monitoring grid may not be uniform within the area of study, the interpolation method, which is similar to that used by the U.S. Environmental Protection Agency, has been shown to capture sufficient spatial variability for the scale of our assessment (Hall et al. 2008).

Our estimates of near-roadway effects also did not account for exposure at school and other locations beside the home, which recent studies suggest may also cause asthma (McConnell et al. 2010). The asthma exacerbation outcomes that we examined vary with regard to their impact on quality of life and resource utilization, ranging from school absences (with relatively modest morbidity for each episode) to hospitalization. It would be useful to evaluate the impact that the alternative risk assessment approach we propose would have on economic costs of air pollution, which preliminary results suggest may be substantial (Brandt et al. 2012).

Although this study focused on the impact of near-roadway exposure on asthma, there is emerging evidence that these exposures also contribute to atherosclerotic heart disease, chronic obstructive pulmonary disease, lung cancer, and childhood neurodevelopmental outcomes (Guxens et al. 2012; HEI 2009; Künzli et al. 2010). Therefore, the implications of near-roadway exposure for the total burden of disease associated with air pollution are potentially quite large. However, the potential increase in physical activity associated with more compact urban design and walkable neighborhoods should also be considered (Saelens et al. 2003). Although there is little evidence that exercise improves asthma, physical activity has known cardiovascular health benefits that could outweigh potential detrimental effects of near-roadway exposure (Giles et al. 2011; Hankey et al. 2012). A more comprehensive assessment of all health-relevant benefits of planned GHG policies would be useful for policy makers. Nonetheless, it is clear that by using available health information to develop “win–win” policies to prevent childhood respiratory disease as cities are redeveloped to reduce GHG emissions, a much larger burden of disease could be prevented.

## Supplemental Material

(131 KB) PDF
