# Supplementary material for: Near-Roadway Pollution and Childhood Asthma: Implications for Developing “Win–Win” Compact Urban Development and Clean Vehicle Strategies
Source: Environ Health Perspect. 2012 Sep 24;120(11):1619–26. doi: 10.1289/ehp.1104785 (PMC3556611; doi:10.1289/ehp.1104785)
Supplement: (131 KB) PDF [file ehp.1104785.s001.pdf]

Online supplement

Near-Roadway Pollution and Childhood Asthma: Implications for Developing “Win-Win” Compact Urban Development and Clean Vehicle Strategies

Laura Perez, Fred Lurmann, John Wilson, Manuel Pastor, Sylvia J. Brandt, Nino Künzli, Rob McConnell

Supplemental Material, Table S1: Yearly number of childhood asthma-related exacerbations attributable to dispersion-modeled near-roadway pollution in combination with reduction of regional NO_2 (top) and regional O_3 (bottom) above background levels in clean communities (scenario 1, dispersion-modeled NOx model) (95% confidence intervals in parentheses)[a].

	Estimated number of exacerbations	Exacerbations due to regional air pollution among children with asthma caused by…			Exacerbations due to other causes among children with asthma	Total
		Near source traffic pollution	caused by near source traffic pollution	all causes	caused by near source traffic pollution	
**NO_2**						
Bronchitis episodes	124034	8000 (1030, 15890)	57100 (19500, 82700)	65100 (22500, 92800)	7250 (1420, 15800)	72370 (34840, 96960)
	100%	6.4% (0.8%, 12.8%)	46.1% (15.7%, 66.7%)	52.5% (18.2%, 74.8%)	5.8% (1.1%, 12.7%)	58.4% (28.1%, 78.2%)
Hospital admissions	3131	45 (10, 80)	330 (255, 405)	375 (295, 450)	340 (80, 585)	715 (440, 965)
	100%	1.5% (0.3%, 2.6%)	10.5% (8.1%, 13.0%)	12.0% (9.4%, 14.4%)	10.8% (2.5%, 18.6%)	22.8% (14.1%, 30.8%)
Emergency room visits	18658	50 (5, 115)	355 (65, 645)	405 (75, 725)	2245 (520, 3840)	2645 (905, 4265)
	100%	0.3% (0.0%, 0.6%)	1.9% (0.3%, 3.5%)	2.2% (0.4%, 3.9%)	12.0% (2.8%, 20.6%)	14.2% (4.8%, 22.9%)
Doctor visits	240696	1240 (100, 2880)	8800 (1800, 15800)	10100 (2000, 17900)	28350 (6630, 48540)	38440 (15460, 59790)
	100%	0.5% (0.0%, 1.2%)	3.7% (0.7%, 6.6%)	4.2% (0.8%, 7.4%)	11.8% (2.8%, 20.2%)	16.0% (6.4%, 24.8%)
**O_3**						
Bronchitis episodes	124034	2310 (0, 5500)	16500 (510, 30900)	18790 (590, 34910)	12900 (3000, 22800)	31700 (12400, 48300)
	100%	1.9% (0.0%, 4.4%)	13.3% (0.4%, 24.9%)	15.1% (0.5%, 28.1%)	10.4% (2.4%, 18.3%)	25.6% (10.0%, 39.0%)
Hospital admissions	3131	2.8 (0.5, 5.6)	19.9 (9.5, 30.6)	22.6 (10.9, 34.3)	380 (90, 650)	400 (110, 680)
	100%	0.1% (0.0%, 0.2%)	0.6% (0.3%, 1.0%)	0.7% (0.3%, 1.1%)	12.2% (2.8%, 20.9%)	12.9% (3.5%, 21.7%)
Emergency room visits	18658	16 (4, 31)	116 (72, 162)	133 (84, 181)	2280 (530, 3900)	2410 (660, 4040)
	100%	0.1% (0.0%, 0.2%)	0.6% (0.4%, 0.9%)	0.7% (0.4%, 1.0%)	12.2% (2.8%, 20.9%)	12.9% (3.5%, 21.6%)
Doctor visits	240696	85 (9, 192)	607 (154, 1072)	692 (175, 1207)	29500 (6800, 50500)	30200 (7400, 51200)
	100%	0.04% (0.004%, 0.1%)	0.3% (0.1%, 0.4%)	0.3% (0.1%, 0.5%)	12.3% (2.8%, 21.0%)	12.5% (3.1%, 21.3%)
Missed school days for respiratory diseases	1350391	41100 (2700, 99400)	288800 (41500, 537300)	329900 (47500, 612100)	126600 (32100, 233100)	456500 (183100, 715400)
	100%	3.0% (0.2%, 7.4%)	21.4% (3.1%, 39.8%)	24.4% (3.5%, 45.3%)	9.4% (2.4%, 17.3%)	33.8% (13.6%, 53.0%)

[a] Reduction in cases is represented by positive values

Supplemental Material, Table S2: Yearly number of childhood asthma-related exacerbations attributable to air pollution (scenario 2, dispersion-modeled NOx model) (95% confidence intervals in parentheses) [a]

	Estimated number of exacerbations	Exacerbations due to regional air pollution among children with asthma caused by…			Exacerbations due to other causes among children with asthma	Total
		Near source traffic pollution	caused by near source traffic pollution	all causes	due to near source traffic pollution	
**NO_2 (O_3 for school absences)**						
Bronchitis episodes	124034	470 (50, 1040)	17500 (4800, 29100)	17900 (4900, 29900)	2800 (600, 5000)	20700 (7900, 32600)
	100%	0.38% (0.04%, 0.8%)	14.1% (3.8%, 23.4%)	14.5% (3.9%, 24.1%)	2.2% (0.5%, 4.0%)	16.7% (6.4%, 26.2%)
Hospital admissions	3131	2 (0, 4)	75 (60, 95)	80 (60, 95)	80 (20, 140)	160 (90, 220)
	100%	0.07% (0.01%, 0.1%)	2.4% (1.9%, 3.0%)	2.5% (2.0%, 3.1%)	2.5% (0.6%, 4.5%)	5.1% (3.0%, 7.1%)
Emergency room visits	18658	2 (0, 5)	80 (15, 145)	80 (15, 150)	490 (110, 860)	570 (180, 940)
	100%	0.0% (0.0%, 0.0%)	0.2% (0.2%, 0.2%)	0.2% (0.2%, 0.2%)	2.6% (0.6%, 4.6%)	2.8% (0.8%, 4.8%)
Doctor visits	240696	50 (0, 130)	2010 (400, 3600)	2060 (410, 3700)	6200 (1380, 11010)	8300 (3170, 13360)
	100%	0.02% (0.002%, 0.05%)	0.8% (0.2%, 1.5%)	0.9% (0.2%, 1.5%)	2.6% (0.6%, 4.6%)	3.4% (1.3%, 5.6%)
School absences	1350391	685 (2, 1722)	25480 (1390, 49650)	26160 (1420, 50900)	34700 (1420, 61400)	60800 (24850, 96970)
	100%	0.05% (0.0%, 0.1%)	1.9% (0.1%, 3.7%)	1.9% (0.1%, 3.8%)	2.6% (0.1%, 4.5%)	4.5% (1.8%, 7.2%)

[a] Reduction in cases is represented by positive value
